# Health-related benefits and adverse events associated with yoga classes among participants that are healthy, in poor health, or with chronic diseases

**DOI:** 10.1186/s13030-021-00216-z

**Published:** 2021-10-07

**Authors:** Takakazu Oka, Battuvshin Lkhagvasuren

**Affiliations:** 1 Department of Psychosomatic Medicine, Graduate School of Medical Sciences, Kyushu University, 3-1-1, Maidashi, Higashi-ku, Fukuoka, 812-8582 Japan; 2 Department of Psychosomatic Medicine, International University of Health and Welfare Narita hospital, 852 Hatakeda, Narita, Chiba, 286-8520 Japan

**Keywords:** Yoga, Adverse events, Stress, Quality of life

## Abstract

**Background:**

Our previous study demonstrated that 42% of yoga class participants in Japan had chronic diseases requiring medication. This raises the question as to whether those with chronic diseases would benefit from practicing yoga or if they are at higher risk for specific adverse events compared to healthy individuals receiving the same instruction.

**Methods:**

To address these questions, 328 adults who started practicing yoga for the first time were asked to complete the Profile of Mood States (POMS), Perceived Stress Scale (PSS), and Medical Outcomes Study Short Form 8, standard version (SF-8™) and to record any adverse events on the first day of the yoga class and again three months later. The participants consisted of three groups: a healthy (H) group (*n* = 70), a poor health (PH) group (*n* = 117), and a chronic disease (CD) group (*n* = 141). The degree of subjective symptoms was also compared between the pre- and post-intervention period in the PH and CD groups.

**Results:**

Typically, yoga classes were held once a week for 60–90 min. The programs included asanas, pranayamas, meditation, isometric yoga, and sukshma vyayama. In the PH and CD groups, the POMS tension-anxiety and fatigue scores decreased and the vigor score increased significantly after the first class. Furthermore, PSS scores decreased and the SF-8™ scores increased significantly three months later. The degree of subjective symptoms such as easy fatigability, shoulder stiffness, and insomnia also decreased over three months. Individuals in these groups experienced more frequent adverse events than those in the H group. The PH and CD groups also experienced a greater variety of symptoms, including psychological ones, not reported by the H group. Adverse events were not so serious that participants stopped practicing yoga during the class. About 60% of all participants were highly satisfied with participating in yoga classes.

**Conclusions:**

If yoga classes are conducted with attention to possible adverse events, yoga practice in a yoga studio may have beneficial effects for people with functional somatic symptoms and chronic diseases, as well as healthy participants. These benefits include reductions in perceived stress and uncomfortable symptoms as well as improved mood and quality of life.

## Introduction

Today, yoga is widely practiced to promote health and improve psychological well-being. A considerable number of studies have demonstrated that yoga has health-related benefits in healthy individuals, such as reductions of negative affect, perceived stress, and subjective somatic symptoms, as well as improvement in health-related quality of life [1–3]. Furthermore, cumulative studies have suggested that yoga also has beneficial effects for patients with stress-related diseases such as depressive disorders [4, 5] and those with chronic illnesses such as breast cancer [6–8] and myalgic encephalomyelitis/chronic fatigue syndrome [9–13].

In contrast, adverse events during yoga practice have also been highlighted [14, 15]. In 2015, we conducted a nation-wide survey in Japan to understand the current experiences in yoga classes, specifically to understand the population that participated in yoga classes and the presence of any adverse events that may occur while practicing yoga. In this survey, 2508 participants from 271 yoga classes were enrolled. We found that the mean age of yoga class participants was 59 ± 13 years and the oldest participant was 93 years of age [14]. Furthermore, 54% of participants reported having a chronic illness and 42% of them were undergoing outpatient treatment at a clinic. The main reasons cited for practicing yoga were to improve well-being and to reduce stress and subjective symptoms.

In many yoga classes, healthy participants and those with chronic diseases practice together in the same classes. Furthermore, yoga instructors may teach yoga without any information regarding the participants’ diseases or sufficient medical knowledge on these respective diseases. In contrast, in research settings, the effects of yoga have been assessed using programs developed specifically for those with particular diseases, all participants in the classes were patients with the same disease, and classes were conducted at a hospital to maximize the benefits and minimize adverse effects.

Considering the current situation in general yoga practice, it is important to elucidate whether individuals who have chronic diseases experience the same health-related benefits from yoga when practicing the same program as healthy individuals. Conversely, it would also be informative to assess the potential adverse effects on those with chronic disease because there is serious concern that there may be adverse events seen exclusively in unhealthy participants when practicing at the same level as healthy participants. To address these questions, we investigated health-related outcomes and adverse events among healthy participants, individuals with poor health, and those with chronic illnesses just starting to practice yoga by attending regular yoga classes.

## Participants and methods

### Participants

This study enrolled 407 adults who had not previously practiced yoga and first attended yoga classes taught by certified yoga instructors, i.e., instructors certified as yoga therapists by the Japan Yoga Therapy Society. All participants voluntarily attended yoga classes to practice yoga in their yoga studios (156 studios in total) for the first time and were not paid for this study. Participants who participated in our previous study [14] were not included in this study. The participants were asked to complete questionnaires on the first day of the yoga classes and again three months later. Among those enrolled, we were unable to obtain data from 73 individuals (17.9%) because of discontinuation of class attendance over the three months. We also excluded the data of 6 individuals (1.5%) from the final analysis because we could not determine to which group they should belong, as described below. Thus, we ultimately analyzed the data of 328 participants (31 men and 297 women) who regularly attended the classes at least twice a month. This study was conducted in collaboration with 114 yoga instructors and 156 yoga classes of the Japan Yoga Therapy Society.

### Procedures

Written informed consent was obtained from all participants on the first day of attendance at a yoga class. They were asked before the yoga class started on their first day of class to fill in forms that included the questionnaires, as described in section 3. After the class, they were asked to record any adverse events and to complete some questionnaires again. On the last day of class (three months later), they again filled out the questionnaires and were also asked to mark their overall satisfaction with the yoga class.

Participants were classified into three groups: a healthy (H) group, a poor health (PH) group, and a chronic disease (CD) group. The H group included individuals who had no subjective symptoms and felt healthy, according to their self-reposts. Therefore, they were not medically examined and not on medication at the hospital. The PH group included individuals who had some somatic or psychological complaints which did not require medication, such as easy fatigability, shoulder stiffness, or coldness in the limbs, or whose symptoms turned out to be functional, i.e. there were no abnormal findings on medical tests which could account for their symptoms by their doctors’ physical examinations and medical tests. The CD group included individuals who had chronic diseases and regularly visited clinics and were on medication.

### Questionnaires

**Fig. 1:**
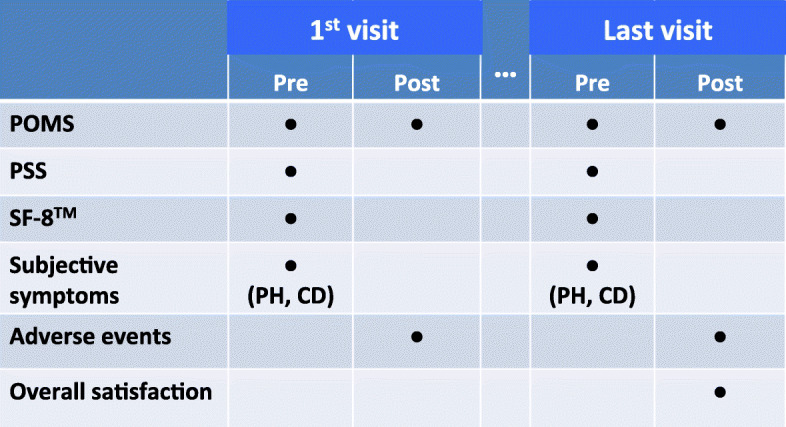
**Schematic representation of the study design.** Each dot represents a questionnaire that participants were asked to complete. Pre: before practicing yoga; Post: after practicing yoga; PH: participants in poor health; CD: participants with chronic diseases. POMS: Profile of Mood States; PSS: Perceived Stress Scale; SF-8™: Medical Outcomes Study Short Form 8, standard version

Participants were asked to complete forms that included the following self-rating questionnaires (Fig. 1).

#### Profile of mood states (POMS)

To assess changes in mood states, the Japanese version of the POMS [16] was used. Because of their limited time before the yoga classes started, they were only asked to answer items relating to vigor (V), fatigue (F), and tension-anxiety (T-A).

#### Perceived stress scale (PSS)

To assess the patient-perceived effects of yoga on stress, the Japanese version of the PSS [17, 18] was administered.

#### Medical outcomes study short form 8, standard version (SF-8™)

To assess the effects of yoga practice for three months on health-related quality of life (QOL), the SF-8™ was administered [19]. The SF-8™ consists of ten subscales, i.e., physical function (PF), role physical (RP), bodily pain (BP), general health perception (GH), vitality (VT), social functioning (SF), role emotional (RE), mental health (MH), physical component summary (PCS), and mental component summary (MCS). The obtained data were scored using norm-based scoring, i.e., scoring based on the national standard value, in which the average score for the Japanese population was set to 50 so that the obtained score could be interpreted by comparing it with the national standard value. Therefore, if a score on one of the subscales was less than 50, it would mean that the function assessed by the subscale was below the average for Japanese people [20].

#### Subjective symptoms

Participants in the PH and CD groups were asked to write down their uncomfortable symptoms and their severities. The degree of the symptom was rated using a numerical rating scale (NRS) from 0 to 10, with 10 being the worst that they could imagine and 0 being nothing.

#### Adverse events

To document adverse events, we created a list of physical and psychological symptoms based on the Cornell Medical Index. Participants were asked to check symptoms that they experienced during the yoga class, similar to how they were assessed in our previous study [14]. In the present study, adverse events were defined as “undesirable symptoms or responses that occurred during a yoga class”, and participants were encouraged to record them.

#### Overall satisfaction

Overall satisfaction with the yoga classes was assessed by marking one of the following, (1) very good, (2) good, (3) neither good nor bad, (4) bad, or (5) very bad, on the last day of the study after the class.

Among these evaluations, the POMS was delivered both before and after the classes on the 1st and last days (after three months) to determine the short-term effects of yoga on mood and fatigue. The PSS and SF-8™, and the form for recording subjective symptoms were distributed just before the class on the 1st and last days. The forms for recording adverse events were distributed after the class on the 1st and last days. The forms for recording overall satisfaction were only distributed after class on the last day.

#### Characteristics of the yoga classes

The yoga instructors were asked to describe in detail their classes, i.e. length of the class, frequency (times per week), and contents of the programs that participants performed.

### Characteristics of the typical yoga class

Typically, the yoga classes were held once a week and the duration was 60 to 90 min. Most classes included 5 to 20 participants. Among the 156 classes, many included individuals from the PH and CD groups, as well as those in the H group. Yoga classes conducted for specific populations, such as yoga classes for breast cancer survivors, were not included in this study. The general idea of each program was made by the Japan Yoga Therapy Society, and classes were conducted based on the society’s textbooks and instructions. Therefore, the basic structure of the classes was similar. However, details of the programs differed according to the instructor and week. Most classes included asanas (regular yoga postures), pranayamas (breathing exercises), meditation, and savasana (corpse pose). These are commonly practiced in every type of yoga class [21]. Characteristics of the programs in these classes included isometric yoga and sukshma vyayama. In some classes, these two were practiced longer than ordinary yoga asanas. Isometric yoga poses are practiced with low impact isometric load with long breathing. As isometric load is applied at the point where joints stop naturally, it does not require a high degree of flexibility or significant stretch. However, it is accompanied with post-isometric muscular relaxation [22, 23]. Therefore, isometric yoga enables muscular relaxation with minimal stretch. This characteristic may be helpful for avoiding any adverse musculoskeletal events, based on our clinical experiences. Sukshma vyayama consists of fine movements of each joint. Sukshma is a Sanskrit word meaning “subtle” and vyayama means “exercise.” Unlike asanas, this exercise does not require strong stretching or muscle contractions that are accompanied by vasoconstriction, but does require continuous attention to proprioception induced by slow movements. The joints of the body are moved slowly and within the physiological range, starting with movement of the distal joints to the proximal joints—that is, first the finger joints are moved, then the wrist joints, elbow joints, and finally the shoulder joints. For example, participants were asked to open and close their fingers and then turn their wrists while paying attention to the movement. Yoga has been reported to help enhance mindful bodily awareness [9, 24–26]. Thus, these programs are aimed at inducing relaxation and enhancing self-awareness rather than improving musculoskeletal fitness. On intervening days without class, participants were encouraged to practice yoga if they could.

### Statistical analyses

Results are presented as the mean ± standard deviation (SD). Differences in categorical variables were tested by Pearson’s chi-square test, whereas differences in continuous variables were assessed by a one-way ANOVA test. A paired *t*-test was used to compare scores between the pre- and post-intervention periods. To avoid the accumulation of type 1 errors across the questionnaire scores and common symptoms, *p* values were adjusted using an adaptive linear step-up procedure that controls for a false discovery rate (FDR). The FDR control at 5% was calculated separately for the questionnaire scores (POMS and SF-8™) and common symptoms to assess group differences in the parameters before and after the intervention. All *p* values of less than 0.05 were considered statistically significant. Effect sizes for significant differences in the questionnaire scores and subjective symptom scores were calculated using Cohen’s *d* with a 95% confidence interval. Effect sizes of 0.20 were considered small, 0.50 moderate, and 0.80 large [27].

### Ethical considerations

This study was conducted with the approval of the ethics committee of Kyushu University. Informed consent was obtained from all participants before the survey was conducted.

## Results

### Participants

This study included 328 total participants: 70 in the H group, 117 in the PH group, and 141 in the CD group. In all groups, most participants were female (90.5% of the total). There was no significant difference in the male:female ratio between groups (*P* = 0.088; *d* = 0.12). Participants were between 21 and 96 years old and the mean age was 54.2 ± 15.0 years (Table 1). There was a significant difference in age between groups (*P* < 0.001; *d* = 0.22). In the CD group, any diseases and the number of participants who suffered from each are indicated in parentheses. Diseases included hypertension (47), low back pain (28), depression (14), autonomic dysfunction (13), climacteric disorder (13), cancer (10), bronchial asthma (9), and type 2 diabetes mellitus (6), among others.

**Table 1:** Demographic characteristics of yoga class attendees

Group	H	PH	CD	Total	***P*** value
**Number**	70	117	141	328	
**Number of females (%)**	59 (84.3)	110 (94.0)	128 (91.4)	297 (90.5)	0.088^*^
**Age range**	26 - 85	21 - 83	23 - 96	21 - 96	
**Mean age**	54.7 ± 14.3	48.5 ± 14.8	58.7 ± 13.9	54.2 ± 15.0	< 0.001^#^

Values are the mean ± standard deviation. The numbers in parenthesis represent the percentage of female participants

*H* healthy participants, *PH* participants in poor health, *CD* participants with chronic diseases

^*^P value was analyzed with the Pearson Chi-Square test

^#^*P* value was analyzed with the One-way ANOVA test

### Effects on vigor, fatigue, and tension-anxiety

To assess the short-term effects of yoga on mood and fatigue, we compared the POMS V, F, and T-A scores before and after practicing yoga on both the 1st and last day (Table 2). The F and T-A scores decreased significantly (*P* < 0.001; *d* = 0.80–1.41) while the V scores increased significantly (*P* < 0.001; *d* = 0.71–1.04) after practicing yoga in both the 1st and last classes in all groups. This suggests that practicing yoga reduced fatigue and tension-anxiety and increased the feeling of energy in all groups, even after just one class.

**Table 2:** Effects of yoga on vigor, fatigue, and tension-anxiety as assessed by the POMS

Group	H					PH					CD				
Subscale	Pre	Post	UPV	FPV	*d*	Pre	Post	UPV	FPV	*d*	Pre	Post	UPV	FPV	*d*
**First**															
**V**	49.6±8.2	56.8±10.7	<0.001	<0.001	0.75	45.9±9.5	56.3±10.4	<0.001	<0.001	1.04	45.5±10.2	53.1±11.4	<0.001	<0.001	0.71
**F**	43.4±8.4	37.7±5.3	<0.001	<0.001	0.80	47.6±9.3	37.5±4.2	<0.001	<0.001	1.41	47.9±11.0	39.6±5.9	<0.001	<0.001	0.94
**T-A**	43.5±7.4	37.0±4.4	<0.001	<0.001	1.07	46.5±10.0	36.6±4.6	<0.001	<0.001	1.27	49.6±11.5	39.0±6.7	<0.001	<0.001	1.12
**Last**															
**V**	49.9±7.7	58.0±9.1	<0.001	<0.001	0.96	48.1±11.4	57.9±10.7	<0.001	<0.001	0.89	47.9±9.4	57.0±10.4	<0.001	<0.001	0.92
**F**	44.2±9.1	37.4±3.7	<0.001	<0.001	0.97	46.1±9.6	37.3±3.9	<0.001	<0.001	1.21	45.9±10.5	38.4±5.3	<0.001	<0.001	0.91
**T-A**	43.2±8.4	36.2±4.0	<0.001	<0.001	1.06	43.8±8.4	37.0±4.8	<0.001	<0.001	0.99	45.7±11.1	37.3±5.7	<0.001	<0.001	0.95

Values are the mean ± standard deviation. *H* healthy participants (*n*=62), *PH* participants in poor health (*n*=108), *CD* participants with chronic diseases (*n*=135). Data from participants that had missing values were excluded from analysis. *Pre* before practicing yoga, *Post* after practicing yoga, *First* the first class, *Last* the last class that was held after three months, *V* vigor, *F* fatigue, *T-A* tension-anxiety, *UPV* Unadjusted *P* values, *FPV* FDR-adjusted *P* values for multiple comparisons (Pre vs Post, paired t-test), *d* effect size between Pre and Post (Cohen’s *d*)

### Effects on perceived stress

**Fig. 2:**
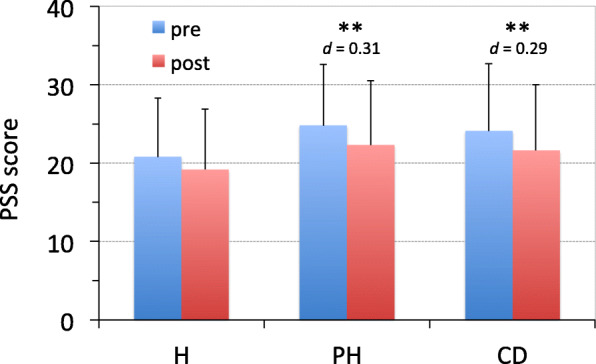
**Effects of yoga on perceived stress as assessed by the PSS.** The PSS scores were compared before (pre) and after (post) practicing yoga for three months in a healthy group (H, *n* = 64), a poor health group (PH, *n* = 111), and a chronic disease group (CD, *n* = 130). Data from participants that had missing values were excluded from analysis. ** *P* < 0.01 (pre vs post, paired *t*-test). PSS: Perceived Stress Scale; H: healthy participants; PH: participants in poor health; CD: participants with chronic diseases. The bar represents standard deviation. *d*: effect size between pre and post (Cohen’s *d*).

To assess the effects of regular yoga practice for three months on perceived stress, we compared the PSS scores before and three months after attending yoga classes (Fig. 2). The PSS score in the H group did not decrease significantly over the three months. However, the PSS scores in the PH and CD groups were reduced significantly (*P* < 0.01; *d* = 0.29–0.31) after three months and approached the pre-value of the H group. This suggests that perceived stress levels in the PH and CD groups were reduced by practicing yoga for three months, whereas the H group did not show a significant change.

### Effects on health-related quality of life

To assess the effects of regular yoga practice for three months on health-related QOL, we compared the SF-8™ subscale scores before (first) and three months after attending the yoga classes (last; Table 3). In the H group, at baseline, all subscale scores were around 50, an average value for the Japanese population, while three subscale scores, GH (general health perception), MH (mental health), and MCS (mental component summary) scores, had increased significantly (*P* < 0.05; *d* = 0.32–0.35) three months later. In contrast, in the PH and CD groups, all subscale scores were below 50 at baseline. However, all 10 subscale scores increased significantly after three months of yoga practice (*P* < 0.05; *d* = 0.27–0.54), except for SF in the CD group (*P* = 0.058; *d* = 0.22), and approached 50. This suggests that the health-related quality of life in the H group was similar to the average Japanese population since the beginning. In contrast, the quality of life in the PH and CD groups was lower than the Japanese average before practicing yoga, and improved such that it approached the Japanese average in all dimensions after practicing yoga for three months.

**Table 3:** Effects of yoga on health-related quality of life as assessed by the SF-8^TM^

Group	H					PH					CD				
Subscale	First	Last	UPV	FPV	*d*	First	Last	UPV	FPV	*d*	First	Last	UPV	FPV	*d*
**PF**	50.6±5.2	50.2±4.4	0.541	0.601	0.08	46.2±8.4	48.9±6.0	0.004	0.007	0.38	43.3±8.8	45.8±8.5	0.005	0.009	0.29
**RP**	49.7±6.0	49.6±5.3	0.883	0.883	0.02	44.7±8.2	46.8±6.6	0.017	0.026	0.29	43.3±8.4	46.0±8.1	0.001	0.003	0.32
**BP**	51.2±7.5	51.7±6.9	0.604	0.647	0.08	43.6±7.8	47.5±7.2	<0.001	<0.001	0.51	43.9±8.3	46.6±8.8	<0.001	0.002	0.32
**GH**	50.3±4.9	52.0±6.0	0.027	0.037	0.32	44.4±8.0	47.8±6.9	<0.001	0.002	0.45	43.3±7.6	47.4±7.4	<0.001	<0.001	0.53
**PCS**	50.1±5.6	49.8±5.4	0.675	0.699	0.05	44.3±7.8	47.0±6.5	0.003	0.006	0.38	42.1±7.8	44.9±8.1	<0.001	0.002	0.34
**VT**	49.4±6.0	50.6±5.5	0.095	0.114	0.21	45.4±6.5	48.7±6.0	<0.001	<0.001	0.54	45.1±6.1	47.4±6.5	0.001	0.002	0.36
**SF**	49.1±8.2	51.0±6.9	0.081	0.101	0.25	44.4±8.7	46.9±7.9	0.002	0.004	0.31	44.5±9.6	46.5±8.6	0.045	0.058	0.22
**RE**	48.6±5.6	49.7±5.4	0.198	0.228	0.19	45.0±7.0	47.4±6.2	0.001	0.002	0.35	45.2±8.0	47.4±6.9	0.003	0.006	0.30
**MH**	48.3±6.1	50.4±5.7	0.021	0.029	0.34	43.3±8.4	47.1±7.8	<0.001	<0.001	0.47	44.3±8.6	47.4±7.8	<0.001	<0.001	0.39
**MCS**	47.2±6.8	49.5±6.3	0.011	0.018	0.35	43.9±8.8	46.7±7.8	<0.001	0.002	0.34	45.2±9.0	47.4±7.8	0.004	0.008	0.27

Values are the mean ± standard deviation. H: healthy participants (*n*=69); *PH* participants in poor health (*n*=117), *CD* participants with chronic diseases (*n*=139). Data from participants that had missing values were excluded from analysis. *First* the first yoga class, *Last* the last yoga class, *PF* physical function, *RP* role physical, *BP* bodily pain, *GH* general health perception, *VT* vitality, *SF* social functioning, *RE* role emotional, *MH* mental health, *PCS* physical component summary, *MCS* mental component summary, *UPV* Unadjusted *P* values, *FPV* FDR-adjusted *P* values for multiple comparisons (First vs Last, paired t-test), *d* effect size between Pre and Post (Cohen’s *d*)

### Changes in degree of subjective symptoms in the PH and CD groups

To assess the effects of regular yoga practice on physical or psychological symptoms or discomforts in the PH and CD groups, we compared the degree of symptoms before (first) and three months after attending the yoga classes (last) using the NRS (Table 4). In the PH group, symptoms noted by at least 10% of the participants included easy fatigability (*n* = 39, 34.2%), shoulder stiffness (*n* = 35, 30.7%), low back pain (*n* = 30, 26.3%), coldness in the body (*n* = 24, 21.1%), headache (*n* = 15, 13.2%), and insomnia (*n* = 14, 12.3%). The degree of these symptoms was reduced significantly (*P* < 0.01; *d* = 0.74–1.46) after three months of yoga practice. In the CD group, symptoms noted by at least 10% of the participants included easy fatigability (*n* = 32, 23.0%), low back pain (*n* = 26, 18.7%), knee joint pain (*n* = 19, 13.7%), shoulder stiffness (n = 19, 13.7%), anxiety (*n* = 18, 12.9%), and insomnia (*n* = 14, 10.1%). The degree of these symptoms also decreased significantly (*P* < 0.05; *d* = 0.54–1.01) after three months of yoga practice.

**Table 4:** Common symptoms by the PH and CD groups

NRS score								
Symptoms	N	%	First	Last	UPV	FPV	***d***	(n)
**PH group**								
**Easy fatigability**	39	34.2	6.5±2.2	4.5±2.4	0.001	0.004	0.87	(30)
**Shoulder stiffness**	35	30.7	7.1±2.3	4.9±2.9	0.001	0.004	0.85	(26)
**Low back pain**	30	26.3	6.0±1.8	4.4±2.6	0.006	0.013	0.74	(23)
**Coldness**	24	21.1	7.8±1.5	5.5±2.1	<0.001	0.002	1.21	(13)
**Headache**	15	13.2	5.7±2.7	2.5±2.5	0.006	0.013	1.23	(11)
**Insomnia**	14	12.3	7.6±1.5	4.7±2.4	<0.001	0.002	1.46	(13)
**CD group**								
**Easy fatigability**	32	23.0	6.6±2.3	4.9±1.6	0.010	0.019	0.86	(23)
**Low back pain**	26	18.7	5.7±2.4	4.3±2.8	0.001	0.004	0.54	(23)
**Knee joint pain**	19	13.7	6.4±2.4	4.6±2.8	0.022	0.036	0.67	(17)
**Shoulder stiffness**	19	13.7	7.1±2.1	4.6±2.8	0.003	0.008	1.01	(16)
**Anxiety**	18	12.9	7.3±1.7	5.4±2.2	0.002	0.005	0.98	(14)
**Insomnia**	14	10.1	7.3±2.2	6.2±2.3	0.040	0.057	0.50	(10)

Values are the mean ± standard deviation. *PH* participants in poor health (*n*=114), *CD* participants with chronic diseases (*n*=139), *First* the first yoga class, *Last* the last yoga class. The degree of symptoms is shown by a numerical rating scale (NRS) with 10 indicating the worst. N illustrates the number of participants who complained of any symptom. The number in parenthesis is the number of participants who scored their degree of symptoms in both the first and the last periods. As data from participants that had missing values were excluded from analysis, statistical analyses were conducted using the number in the parentheses. Multiple symptoms were included. Unadjusted *P* values. *FPV* FDR-adjusted *P* values for multiple comparisons (First vs Last, paired t-test) *d* effect size between Pre and Post (Cohen’s *d*)

### Adverse events

Adverse events that occurred during the first and last classes were recorded just after each lesson. Generally, adverse events were more frequent in the PH and CD groups than the H group, and the incidence during the last class was less than during the first class (Table 5). Among participants in the H, the PH, and the CD groups, any adverse events were reported by 28.6, 33.3, and 41.1%, respectively, during the first class and 22.9, 26.5, and 36.2%, respectively, during the last class. Symptoms experienced by more than 5.0% of participants included faintness (*n* = 4, 5.7%) and dizziness (n = 4, 5.7%) in the H group, joint pain (*n* = 7, 6.0%), muscular pain (*n* = 8, 6.8%), faintness (n = 7, 6.0%), twitching (*n* = 6, 5.1%), and dizziness (n = 6, 5.1%) in the PH group, and coughing (n = 8, 5.7%), runny nose (n = 6, 5.1%), joint pain (*n* = 11, 7.8%), muscular pain (*n* = 17, 12.1%), feeling hot/cold (*n* = 10, 7.1%), faintness (*n* = 9, 6.4%), and dizziness (*n* = 14, 9.9%) in the CD group. Among symptoms that none of the participants in the H group reported, the PH group experienced 7 symptoms, such as numbness (*n* = 5, 4.2%), epigastric and abdominal pain, excessive perspiration, anxiety (*n* = 2, 1.7%, respectively), breathlessness, confusion, and recollection of bad experiences (*n* = 1, 0.9%, respectively). Among symptoms that none of the participants in the H group reported, the CD group experienced 13 symptoms, such as tension, recollection of bad experiences (*n* = 4, 2.8%, respectively), breathlessness, epigastric and abdominal pain, excessive perspiration (*n* = 3, 2.1%, respectively), pruritus, numbness, confusion, anxiety (*n* = 2, 1.4%, respectively), chest pain, diarrhea, urge to cry, and irritation (*n* = 1, 0.7%, respectively). In the CD group, adverse symptoms reported by participants of each disease were described in the Table 6. These adverse events were mild and there were no serious adverse events that caused a participant to quit practicing yoga or that required medical treatment.

**Table 5:** Adverse events experienced by participants during yoga practice

	Group	H		PH		CD	
Systems	Symptoms	First	Last	First	Last	First	Last
**Eyes and ears**	**Blackout**	0	0	0	3	2	1
	**Pruritus of the eye**	2	0	1	1	1	2
	**Tinnitus**	1	1	2	1	2	2
**Respiratory**	**Coughing**	2	1	3	2	**6**	**8**
	**Congested nose**	1	1	2	3	3	2
	**Runny nose**	1	3	0	3	**6**	2
	**Sputum production**	2	0	2	2	0	3
**Cardiovascular**	**Chest pain**	0	0	0	0	1	0
	**Palpitation**	2	0	1	3	3	1
	**Breathlessness**	0	0	0	1	3	1
**Gastrointestinal**	**Nausea**	1	2	0	0	2	0
	**Epigastric and abdominal pain**	0	0	2	1	2	3
	**Diarrhea**	0	0	0	0	1	0
**Musculoskeletal**	**Joint pain**	2	1	**7**	3	**8**	**11**
	**Muscular pain**	3	0	5	**8**	**9**	**17**
**Skin**	**Flushing of the face**	3	1	2	0	3	3
	**Excessive perspiration**	0	0	2	1	3	1
	**Pruritus of the skin**	0	0	0	0	2	2
**Neurological**	**Headache**	1	2	5	2	2	4
	**Heaviness of the head**	0	2	1	3	2	0
	**Feeling hot and cold**	0	0	4	2	**10**	2
	**Faintness (daze)**	**4**	3	**7**	4	**9**	**8**
	**Numbness of a certain body part**	0	0	5	2	2	1
	**Twitching in a certain body part**	1	2	**6**	2	5	4
	**Dizziness**	2	**4**	**6**	5	**14**	5
**Fatigue**	**Feeling of exhaustion**	1	1	3	2	5	3
	**Feeling sick**	0	1	0	1	1	0
**Psychological**	**Tension**	0	0	0	0	4	3
	**Confusion**	0	0	1	0	2	2
	**Urge to cry**	0	0	0	0	1	0
	**Anxiety**	0	0	2	1	2	0
	**Irritation**	0	0	0	0	1	0
	**Shaking of the body**	0	1	0	0	1	0
	**Recollection of bad experiences**	0	0	1	1	4	2
**The number experiencing a symptom** (%)		20 (28.6)	16 (22.9)	39 (33.3)	31 (26.5)	58 (41.1)	51 (36.2)
**The number of valid responses**		70		117		141	

*H* healthy participants (*n*=70), *PH* participants in poor health (*n*=117), *CD* participants with chronic diseases (*n*=141). Data from participants that had missing values were excluded from analysis. *First* the first yoga class, *Last* the last yoga class. Symptoms reported by more than 5.0% of participants in each group are shown in bold (First vs Last, paired t-test)

**Table 6:** Adverse events experienced by participants with chronic diseases during yoga practice

Systems	Symptoms	Hypertension		Low back pain		Depression		Autonomic dysfunction		Climacteric disorder		Cancer		Bronchial asthma		Diabetes mellitus	
		First	Last	First	Last	First	Last	First	Last	First	Last	First	Last	First	Last	First	Last
**Eyes and ears**	**Blackout**	1	0	0	0	1	0	0	0	0	0	0	0	0	**1**	0	0
	**Pruritus of the eye**	0	1	0	0	0	0	0	0	0	0	**1**	0	0	0	0	0
	**Tinnitus**	0	1	1	0	0	0	0	1	0	0	0	0	0	0	0	0
**Respiratory**	**Coughing**	0	3	2	1	0	1	0	**2**	0	0	**1**	**1**	0	**1**	0	0
	**Congested nose**	1	0	0	0	0	0	0	0	0	0	**1**	0	**1**	0	0	0
	**Runny nose**	1	0	2	1	1	0	1	0	**2**	0	0	**1**	0	0	0	0
	**Sputum production**	0	1	0	1	0	0	0	0	0	0	0	0	0	**1**	0	0
**Cardiovascular**	**Chest pain**	0	0	0	0	0	0	0	0	0	0	0	0	0	0	0	0
	**Palpitation**	0	0	0	0	1	1	**2**	1	1	0	0	0	0	0	0	0
	**Breathlessness**	0	0	2	1	1	0	0	0	0	0	0	0	0	0	0	0
**Gastrointestinal**	**Nausea**	1	0	0	0	0	0	0	0	0	0	0	0	**1**	0	0	0
	**Epigastric & abdominal pain**	0	2	1	1	**2**	0	1	0	1	**2**	0	0	0	0	0	0
	**Diarrhea**	0	0	0	0	0	0	0	0	0	0	0	0	0	0	0	0
**Musculoskeletal**	**Joint pain**	4	4	2	**4**	1	**2**	0	**2**	0	0	0	**1**	0	0	0	0
	**Muscular pain**	2	**6**	1	2	**2**	**2**	1	**3**	1	1	0	**3**	0	0	**1**	0
**Skin**	**Flushing of the face**	1	1	1	0	0	1	0	1	0	0	**1**	0	0	0	0	0
	**Excessive perspiration**	3	0	0	0	0	0	**2**	0	0	0	0	0	0	0	**1**	**1**
	**Pruritus of the skin**	1	1	1	0	1	0	1	0	1	0	0	0	0	0	0	0
**Neurological**	**Headache**	1	1	0	**3**	0	1	0	0	0	1	**1**	0	0	0	0	0
	**Heaviness of the head**	1	0	1	0	0	0	0	0	0	0	0	0	0	0	0	0
	**Feeling of hot and cold**	1	1	1	0	**2**	0	**3**	0	**3**	0	0	0	0	0	**1**	0
	**Faintness (daze)**	2	4	1	**3**	**2**	1	1	**2**	1	1	0	0	0	**1**	0	0
	**Numbness of a certain body part**	0	1	0	0	1	0	1	1	0	0	0	0	0	0	0	0
	**Twitching in a certain body part**	2	1	0	0	**2**	1	**2**	1	0	0	0	0	**1**	**1**	0	0
	**Dizziness**	2	2	2	1	**3**	0	**3**	0	1	1	**1**	0	**2**	**3**	0	0
**Fatigue**	**Feeling of exhaustion**	2	2	0	0	1	0	0	0	1	0	**1**	**2**	0	0	0	0
	**Feeling sick**	1	0	0	0	0	0	0	0	0	0	0	0	0	0	0	0
**Psychological**	**Tension**	2	0	1	0	0	1	1	1	1	0	0	0	**1**	0	0	**1**
	**Confusion**	0	0	1	0	0	0	1	0	1	0	0	**1**	0	0	0	0
	**Gloominess**	0	0	0	0	0	0	0	0	0	0	0	0	0	0	0	0
	**Urge to cry**	0	0	0	0	0	0	0	0	0	0	0	0	**1**	0	0	0
	**Anxiety**	1	0	0	0	0	0	1	0	0	0	0	0	**1**	0	0	0
	**Irritation**	1	0	0	0	0	0	0	0	0	0	0	0	0	0	0	0
	**Shaking of the body**	0	0	0	0	0	0	0	0	0	0	0	0	**1**	0	0	0
	**Recollection of bad experience**	1	0	1	1	1	0	**2**	1	0	0	0	0	**1**	0	0	0
	**Scary thoughts**	0	0	0	0	0	0	0	0	0	0	0	0	0	0	0	0
	**Frightened feeling**	0	0	0	0	0	0	0	0	0	0	0	0	0	0	0	0
	**Heightened emotion**	0	0	0	0	0	0	0	0	0	0	0	0	0	0	0	0
**The number of attendees who had some the above symptoms (%)**		16 (34.0)	18 (38.3)	11 (39.3)	8 (28.6)	10 (71.4)	5 (35.7)	10 (76.9)	7 (53.8)	6 (46.2)	3 (23.1)	4 (40.0)	5 (50.0)	2 (22.2)	4 (44.4)	3 (50.0)	1 (16.7)
**The number of valid responses**		47		28		14		13		13		10		9		6	

Data from participants that had missing values were excluded from analysis. First: the first yoga class; Last: the last yoga class. Symptoms reported by more than 5.0% of participants in each group are shown in bold

### Overall satisfaction and benefits of yoga

**Fig. 3:**
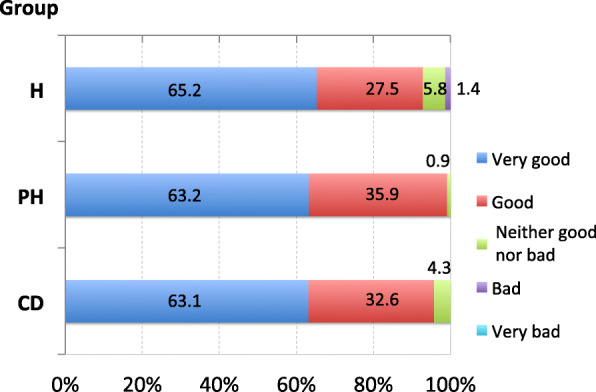
**Overall satisfaction in attending yoga classes.** The numbers in the figure indicate percentage (%).H: healthy participants; PH: participants in poor health; CD: participants with chronic diseases

To assess overall satisfaction, we asked participants whether or not yoga was helpful for maintaining and improving their health by giving a score from 1 to 5: (1) very good, (2) good, (3) neither good nor bad, (4) bad, or (5) very bad. In all groups, more than 60% of participants answered “very good” and more than 90% of those answered “good or very good”. In the H group, 1.4% of participants answered “bad”. However, no one answered “very bad” in any group (Fig. 3).

## Discussion

The aim of this study was to investigate if individuals with poor health or chronic diseases would benefit from yoga in a similar way to healthy individuals and to assess any specific adverse events unhealthy participants might experience when they practice the same yoga program alongside healthy participants.

In our previous study [14], we investigated the characteristics of yoga class participants and yoga-related adverse events. We found that 42% of participants were patients who were on regular medication, and 28% of attendees reported some type of undesirable symptoms. These findings prompted us to conduct the current study, with the aim described above. In this study, we found that participants in the PH and CD groups obtained some benefit from yoga, as did those in the H group. The incidence and characteristics of adverse events in the H group were similar to those in our previous study. However, this study suggested that the frequency of adverse events is higher in the PH and CD groups compared to the H group, and that special attention is necessary when instructing yoga to the PH and CD groups, as these individuals present more variable symptoms than the H group.

### Benefits

First, this study suggests that yoga reduces negative affects and perceived stress while improving health-related QOL in individuals with poor health and chronic diseases. Practicing yoga for three months decreased the POMS T-A and F scores and increased V scores in all groups, suggesting that yoga reduced tension, anxiety, and fatigue and increased energy in all participants. This beneficial effect was obtained even after the first class. Yoga also reduced PSS scores in the PH and CD groups, suggesting that yoga can reduce perceived stress levels among individuals with poor health and with chronic diseases. In contrast, the PSS score did not decrease significantly in the H group after three months. This does not necessarily mean that yoga does not have any effect on perceived stress in healthy individuals; rather, it may be due to lower stress levels in the H group from the outset, as the PSS score of the H group (20.8 ± 7.5) before practicing yoga was lower than that for average Japanese adults (25.4 ± 8.6) [18]. Furthermore, yoga improved health-related QOL in all groups. Among the ten SF-8™ subscale scores, yoga improved all subscale scores in the PH and CD groups, whereas it improved three subscale scores in the H group. This also does not necessarily mean that yoga had little effect on health-related QOL in healthy participants, and instead may be due to a ceiling effect because every SF-8™ subscale score of the H group was around 50, an average value for Japanese people. On the other hand, in the PH and CD groups, all subscale scores were below 50, suggesting that their QOL was worse than the average for Japanese people. Given that after three months of yoga, all of the subscale scores approached the pre-values of the H group, this suggests that yoga may improve their QOL near to that of healthy participants. It is noteworthy that yoga improved all subscale scores in these groups because it suggests that yoga has beneficial effects, both physically and mentally, in individuals with poor health or chronic diseases.

Second, yoga reduced the degree of uncomfortable symptoms in the PH and CD groups. Common symptoms reported in more than 10% of both groups included easy fatigability, shoulder stiffness, low back pain, and insomnia. After three months, the average scores of these symptoms, other than insomnia, decreased significantly in the CD group, as shown in the Table 4.

Taken together, this study suggests that practicing yoga has psychological and physical benefits in individuals with poor health and in those with chronic diseases.

### Adverse events

This study also indicated that the potential for adverse events should be taken into consideration. We demonstrated that the incidence of adverse events on the day of the first class was 28.6% in the H group and that it was more prevalent in the PH group (33.3%) and the CD group (41.1%). In all groups, the percentage of participants experiencing any particular incidence had decreased on the last day. On neither day did anyone have to discontinue the class due to adverse events or any other reason. While the incidence of adverse events may seem rather high to some, these rates were comparable to those found in our previous nation-wide survey on adverse events associated with yoga classes [14]. In a previous study, we investigated the incidence of adverse events during yoga practice among 2508 yoga class participants throughout Japan and found that 27.8% of participants experienced some adverse events after participating in a class. In that study, among participants who had uncomfortable symptoms, 1.9% of them had to discontinue the class [14]. Therefore, the incidence of adverse events in the healthy participants in the present study was almost the same as that of our previous study.

The present study addressed several important points to be considered when yoga instructors teach classes with participants in poor health or those with chronic diseases alongside healthy participants. First, the incidence of adverse events is likely to be more frequent among these individuals than healthy individuals. Second, these participants may experience more varied symptoms than healthy participants because participants of these groups reported numerous symptoms that healthy individuals did not report. For some individuals, it may be difficult to avoid these symptoms because some of them seem to be induced by the activity of yoga itself, i.e., changing autonomic nervous function from a sympathetic-dominant state to a parasympathetic-dominant state [28] [29]. Although this effect is beneficial for reducing stress [30], it is possible that it may cause or exacerbate coughing and runny noses in patients with bronchial asthma and allergic/vasomotor rhinitis, abdominal pain and diarrhea in patients with irritable bowel syndrome, and faintness or dizziness in patients with hypotension, especially orthostatic hypotension because these symptoms are induced or exacerbated by sympathetic inhibition and/or parasympathetic activation. One might wonder if these symptoms are really related to yoga practice. Indeed, symptoms such as coughing, congested nose, or runny nose, were also reported after the yoga classes in our previous nationwide survey [14]. This suggests that these symptoms could be reported after any yoga class, and we believe that that these symptoms could count as adverse events. Therefore, it is important to keep in mind that, although this characteristic of yoga is beneficial for reducing stress in healthy participants, it is possible that it may cause or exacerbate symptoms in patients with diseases in which the parasympathetic nerve is involved in the pathophysiology. Third, yoga may have mixed effects on symptoms of the musculoskeletal system. This study demonstrated that yoga reduced low back pain as a long-term effect. However, as a short-term effect, practicing yoga induced arthralgia and myalgia in more than 5% of participants in the PH and CD groups. Fourth, it is important to remember that these participants may also have psychological symptoms. Generally, yoga is known to induce relaxation and thereby decrease negative affects such as anxiety [3]. However, this study demonstrated that yoga induced negative affects such as tension, confusion and anxiety in a few individuals in these groups. This might be due to “relaxation-induced anxiety” [31, 32]. This contradictory effect can be observed even in healthy individuals. However, this study demonstrated that it could be observed more frequently in participants with health concerns. Another explanation for this might be due to abreaction, i.e. recalling negative experiences from the past during yoga practice. This phenomenon is not uncommon, either, especially in individuals with psychologically traumatic experiences in their past. In people who have suppressed their negative emotions or have had traumatic experiences in the past, a yoga practice that focuses inside the body in a quiet and relaxed manner may enable the person to be aware of their inner sensations, including negative emotions and traumatic memories that the individual previously escaped or had suppressed in their daily life. Lastly, it is important to pay special attention to participants with some medical conditions. Although the number was small, this study suggested some characteristics of adverse symptoms in participants in each disease. For example, participants with depression, autonomic dysfunction, and climacteric disorder may have numerous symptoms with a wide range of systems, whereas participants with cancer may feel exhaustion more easily when they practice yoga together with healthy participants. Therefore, when instructing yoga to these types of participants together with healthy individuals, yoga instructors should be aware of the potential for these adverse events and prepare to minimize them.

### Overall satisfaction

In spite of these adverse events, more than 60% of participants of poor health and those with chronic diseases reported high satisfaction, comparable to that of healthy individuals. These high rates of satisfaction may be due to the long-term beneficial effects, including reduction of negative affect and perceived stress, improvement of health-related QOL, and decrease in degree of uncomfortable symptoms in these individuals, even though they experience some undesirable symptoms, such as muscular pain, faintness, or dizziness (as shown in the Table 5), especially in the beginning as a short-term effect. Furthermore, a considerable number of participants reported that it was nice to have an opportunity to learn practical methods to deal with stress, such as relaxation techniques, breathing methods, or meditation, by attending the yoga class. It is possible that most, if not all, subjective symptoms in the PH group are stress-related and that participants in the CD group feel stress due to the burden of their chronic diseases. These hypotheses may be reasonable because medical treatment, especially medication alone, does not necessarily teach them how to cope with their stress, even though it could alleviate their symptoms.

### Limitations

This study has several limitations to note. First, it is difficult to generalize these findings to other styles of yoga classes because there are many kinds of yoga, and yoga instructors are constantly developing new types of yoga to generate interest in their classes. The characteristics of yoga classes that aligned with the current study were the inclusion of isometric yoga and sukshma vyayama as well as common practices in other yoga classes such as asanas, pranayamas, and meditation. As described earlier, these types of yoga programs would enable participants to obtain post-isometric relaxation and to enhance self-awareness, which may help them find methods for coping with their stress. For example, one participant reported, “I noticed how shallow my breathing was, how I wasn’t relaxed in daily life, and how these things made my conditions uncomfortable. Through practicing yoga, I became able to notice my stress and stress-induced bodily changes quickly, and became able to deal with it in the way I learned in the yoga classes.” Second, 17.9% of participants dropped out during the study. Although the reasons are unclear, it is possible that this caused some bias to the results. In particular, it is possible that beneficial effects and high satisfaction were obtained because the participants were highly motivated. Furthermore, most participants were female (around 90%). Therefore, it is not clear if male participants and those who were less motivated would have similar benefits from yoga. Third, the CD group included a limited number of common chronic diseases, such as hypertension or low back pain. Therefore, future studies are warranted to determine if this finding is also applicable to those with other chronic diseases such as hyperthyroidism, multiple sclerosis, or schizophrenia, and to determine if specific attention should be paid to certain diseases while instructing yoga. Fourth, ages were different among the three groups, with ages ranging from 21 to 96 years old in this study. The effects and adverse events may differ depending on the participant’s age. Fifth, it is also reasonable to suspect that beneficial effects or incidence of adverse events may differ depending on how enthusiastic the participants were about yoga. It also may differ depending on how many times they attended class and how many times they practiced outside of class, or the level/experience of the yoga instructor. To address these questions, more precise analyses are necessary in the future.

## Conclusions

This study investigated if individuals with poor health or chronic diseases would benefit from yoga, similar to healthy individuals, and if they might be prone to experiencing specific adverse events when practicing the same program as healthy participants. The findings showed that many patients received benefit from yoga in terms of reducing negative affects, perceived stress and improvement of health-related QOL. It also suggests that special attention to those in poor health is necessary because their incidence of adverse events will be higher than in healthy participants, especially at the beginning, and they may have a greater variety of adverse symptoms including psychological ones. Nevertheless, more than 60% of the PH and the CD groups reported high satisfaction, which is at least partly due to the benefits described above. Therefore, practicing yoga in classes that include isometric yoga and sukshma vyayama as well as asanas, pranayamas, and meditation, may have potential benefits for individuals with poor health or common chronic illnesses, including reduction of negative affect, perceived stress, and uncomfortable symptoms and improvement of positive affect and health-related QOL if the instruction is conducted with attention to the prevention of adverse events.

## Data Availability

Data sharing is not applicable.

## Abbreviations

BP: bodily pain
CD: chronic disease
F: fatigue
GH: general health perception
H: healthy
MCS: mental component summary
MH: mental health
NRS: numerical rating scale
PCS: physical component summary
PF: physical functioning
PH: poor health
POMS: Profile of mood States
PSS: Perceived Stress Scale
RE: role emotional
RP: role physical
QOL: quality of life
SF: social functioning
SF-8™: Medical Outcomes Study Short Form 8, standard version
T-A: tension-anxiety
V: vigor
VT: vitality
